# Detecting hypoglycemia-induced electrocardiogram changes in a rodent model of type 1 diabetes using shape-based clustering

**DOI:** 10.1371/journal.pone.0284622

**Published:** 2023-05-18

**Authors:** Sejal Mistry, Ramkiran Gouripeddi, Candace M. Reno, Samir Abdelrahman, Simon J. Fisher, Julio C. Facelli

**Affiliations:** 1 Department of Biomedical Informatics, University of Utah, Salt Lake City, Utah, United States of America; 2 Center for Clinical and Translational Science, University of Utah, Salt Lake City, Utah, United States of America; 3 Division of Endocrinology, Metabolism, and Diabetes, Department of Internal Medicine, University of Utah, Salt Lake City, Utah, United States of America; 4 Division of Endocrinology, Diabetes and Metabolism, Department of Internal Medicine, University of Kentucky, Lexington, Kentucky, United States of America; York University, CANADA

## Abstract

Sudden death related to hypoglycemia is thought to be due to cardiac arrhythmias. A clearer understanding of the cardiac changes associated with hypoglycemia is needed to reduce mortality. The objective of this work was to identify distinct patterns of electrocardiogram heartbeat changes that correlated with glycemic level, diabetes status, and mortality using a rodent model. Electrocardiogram and glucose measurements were collected from 54 diabetic and 37 non-diabetic rats undergoing insulin-induced hypoglycemic clamps. Shape-based unsupervised clustering was performed to identify distinct clusters of electrocardiogram heartbeats, and clustering performance was assessed using internal evaluation metrics. Clusters were evaluated by experimental conditions of diabetes status, glycemic level, and death status. Overall, shape-based unsupervised clustering identified 10 clusters of ECG heartbeats across multiple internal evaluation metrics. Several clusters demonstrating normal ECG morphology were specific to hypoglycemia conditions (Clusters 3, 5, and 8), non-diabetic rats (Cluster 4), or were generalized among all experimental conditions (Cluster 1). In contrast, clusters demonstrating QT prolongation alone or a combination of QT, PR, and QRS prolongation were specific to severe hypoglycemia experimental conditions and were stratified heartbeats by non-diabetic (Clusters 2 and 6) or diabetic status (Clusters 9 and 10). One cluster demonstrated an arrthymogenic waveform with premature ventricular contractions and was specific to heartbeats from severe hypoglycemia conditions (Cluster 7). Overall, this study provides the first data-driven characterization of ECG heartbeats in a rodent model of diabetes during hypoglycemia.

## Introduction

Hypoglycemia occurs when blood glucose levels drop below 70 mg/dL (3.89 mmol/L) and is common among individuals with insulin-treated diabetes [1, 2]. When severe, hypoglycemia can lead to several complications, including sudden death [3–5]. Sudden death related to hypoglycemia may present as "dead in bed syndrome," where individuals with insulin-treated diabetes experience nocturnal severe hypoglycemia and are unable to seek life-saving treatment to correct hypoglycemia [6, 7]. Sudden death is a leading cause of mortality in children with type 1 diabetes, with an estimated incidence of 8–17% [8, 9]. Though the precise mechanisms of hypoglycemia-induced mortality are unclear, dysregulation of cardiac electrical signals are thought to play a role. A clearer understanding of the cardiac changes associated with hypoglycemia-induced mortality are needed to better understand the pathogenesis of "dead-in-bed syndrome," identify patients at risk for hypoglycemia-induced arrhythmias, and ultimately prevent death.

Clinical studies have identified abnormal electrocardiogram (ECG) changes during severe hypoglycemia in patients with insulin-treated diabetes, including QT interval prolongation, abnormal T-wave morphology, bradycardia, and other cardiac arrhythmias [7, 10–13]. Though death associated with hypoglycemia and cardiac arrhythmias have been documented [14, 15], a causal link between hypoglycemic-induced arrhythmias has been difficult to ascertain in human studies for several reasons. Simultaneous electrophysiologic and glucose monitoring is rarely performed in clinical practice and the induction of severe hypoglycemia in individuals with insulin-treated diabetes is dangerous and unethical. Therefore, rat models of diabetes are widely used to understand electrophysiologic dysfunction during hypoglycemia [16]. Though rats have slight ECG parameters that are distinct from human ECGs (lack of a detectable Q wave, faster heart rate, T-wave onset continuous with S waves, etc.) [17], previous studies indicate that rat ECGs share many of the morphologic changes commonly observed in hypoglycemic humans [18, 19].

Previous work demonstrated a pattern of QT interval prolongation, premature atrial contractions, premature ventricular contractions, and first-degree heart block following induction of hypoglycemia in insulin-injected non-diabetic rats [18]. When compared to non-diabetic rats, diabetic rats had a two-fold increase in mortality and increased frequency of first-degree heart block and second-degree heart block during hypoglycemia [19]. Together, these findings suggest that mortality in diabetic rats is attributed to increased frequency of specific arrhythmic patterns during severe hypoglycemia. Analysis of individual morphologic changes comparing the intersection of diabetes status, mortality, and glycemic level may provide further insight into hypoglycemia-induced mortality. However, these analyzes were not conducted due to the complexity of performing detailed analyses in large datasets using traditional statistical methods.

Machine learning methods are well-equipped to identify complex electrical patterns in large datasets. These methods may also help identify novel morphologic changes associated with diabetes status, death status, and glycemic level. Several machine learning approaches have been implemented in human studies to classify different types of arrhythmias, detect novel longitudinal changes in ECGs, and identify heartbeats in noisy data [20–23], but have not been tested in rat models of hypoglycemia-induced mortality.

Therefore, this work aims to identify distinct patterns of ECG heartbeat changes that correlate with glycemic level, diabetes status, and mortality using a rodent model. We describe a novel method for segmenting heartbeats from rodent ECGs and train a shape-based unsupervised clustering algorithm to identify data-driven subgroups of heartbeat morphology. Heartbeats representing cluster centers were identified and evaluated for morphologic and phenotypic features. This work concludes with a discussion of relevant findings and limitations.

## Materials and methods

**Fig 1:**
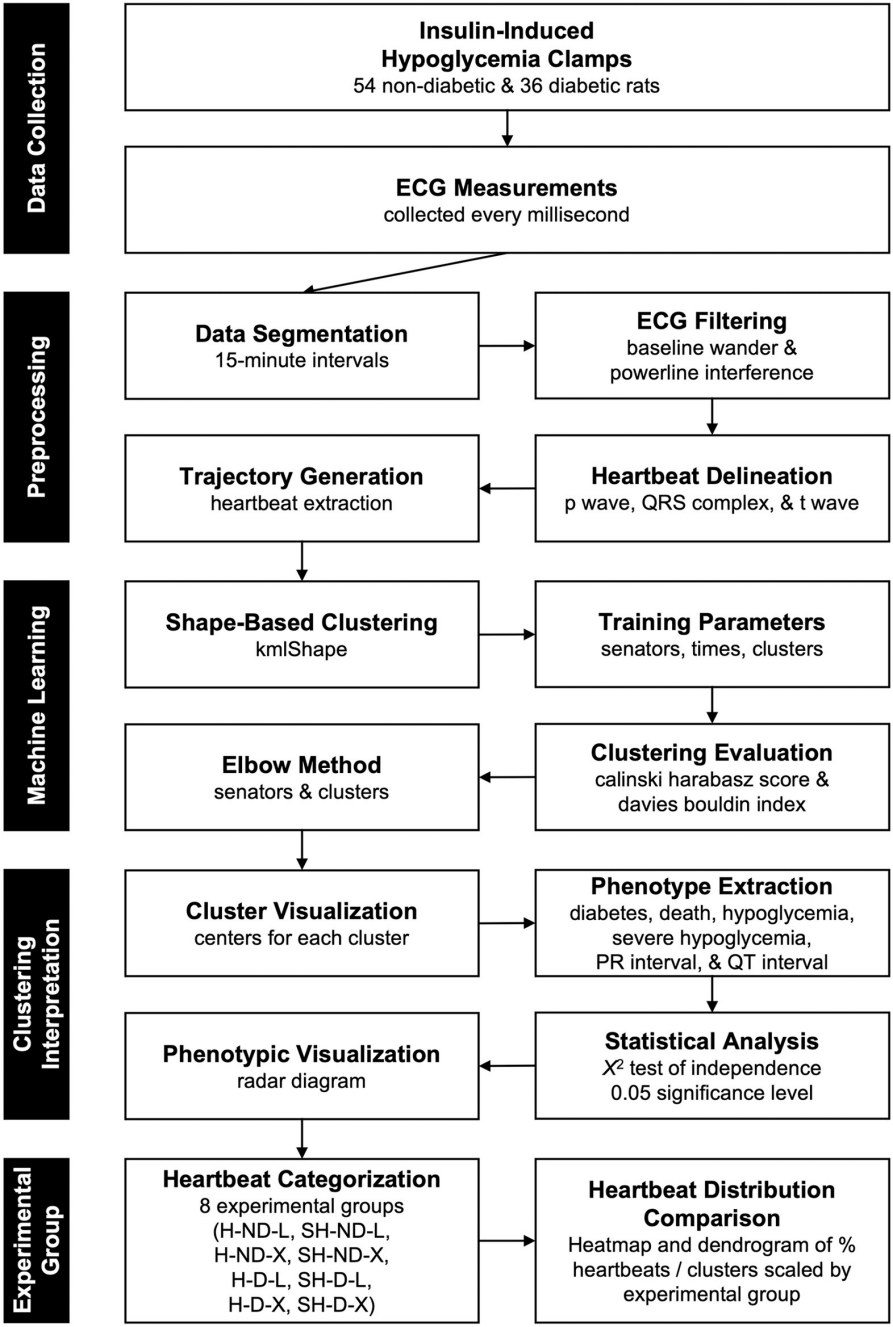
Summary of analytical workflow. The data collection, various preprocessing techniques, unsupervised machine learning algorithm, clustering evaluation, and analysis by experimental group are detailed.

An overview of the analytical methods used in this project is summarized in Fig 1. Experimental studies were done in accordance with and approved by the Animal Studies Committee at the University of Utah School of Medicine and computational analyses were performed using the Center for High Performance Computing at the University of Utah in python (v3.7.10) and R (v4.0.2).

### Data collection

Retrospective data was obtained from studies conducted by Reno et al. [3, 18, 19, 24]. Adult male Sprague-Dawley rats (weight, 250–300 g; Charles River Laboratories, Malvern, PA) were housed individually in temperature- and light-controlled environments and fed ad libitum a standard chow diet and water. Rats underwent surgery for carotid artery and jugular vein cannulation as previously described [18]. ECG leads were also placed subcutaneously during surgery. One wire was placed over the lower left rib cage and one was placed over the right supraclavicular fossa. A reference wire was placed over the back. Two days after cannulation, one set of rats received intraperitoneal injections of streptozotocin (65 mg/kg; Sigma, St. Louis, MO) to induce diabetes (n = 37). A second group of rats received sodium citrate buffer and acted as a control, non-diabetic group (n = 54). Blood glucose was measured from the tail vein with a glucometer (Ascensia Contour; Bayer HealthCare, Mishawaka, IN) to ensure diabetes. Rats were diabetic for two weeks prior to the hypoglycemic clamp.

Overnight fasted, awake, unrestrained non-diabetic and diabetic rats underwent hyperinsulinemic (0.2 units ⋅ kg−1 ⋅ min−1; Humulin R), severe hypoglycemic (10–15 mg/dL) single step clamps with continuous ECGs for 3 hours, as previously described [18]. ECGs were recorded every millisecond (ms) using PowerLab 26T software (LabChart; ADInstruments, Colorado Springs, CO). Arterial blood was measured to determine glucose levels during the clamp with a glucometer (Ascensia Contour BG monitors; Bayer Healthcare Mishawaka, IN) every 15-minutes.

Animals were sacrificed under isoflurane according to the protocols of the IACUC at the University of Utah. During surgery, animals were given ketamine/xylazine to induce an anesthetic plane. Animals were monitored during surgery. Afterward, animals were given rimadyl for several days to minimize pain. Any animal that showed signs of sickness, lethargy, or were overall not doing well, was sacrificed.

### Preprocessing

ECG data was first segmented into 15-minute intervals that aligned with collection of blood glucose measurements. All ECG segments were then filtered for baseline wander [25, 26] and powerline interference [27] with a notch and band-pass butterworth filter, respectively (Python, Heart Rate Analysis Toolkit, v0.8.1 [28, 29], S1A Fig in S1 File) and are further described in S1 Methods in S1 File.

Next, heartbeat delineation was performed to identify R, P, and T waves in each ECG segment for each rat (S1B Fig in S1 File). R waves were first identified using a peak-finding algorithm (Python, SciPy, v1.7.3 [30]) by estimating the prominence, width, and relative height of each peak and is further described in S1 Methods in S1 File. For each identified R peak, a sliding window method was used to identify the corresponding P and T waves and is further described in S1 Methods in S1 File. All identified heartbeats were then scaled using a min-max scaler (Python, scikit-learn, v0.24.2 [31]) and missing values were imputed using the mean of the trajectory (R, longitudinalData, v2.4.1 [32, 33]).

### Unsupervised machine learning

kmlShape, a longitudinal time-series clustering algorithm [32], was used to identify distinct subgroups of extracted heartbeats. kmlShape utilizes the Fréchet distance [34] for shape-respecting partitioning, which allows for clustering based on ECG heartbeat morphology rather than the specific timing of electrical changes alone. Because use of the Fréchet distance increases computational complexity, data reduction methods were applied to reduce the number of trajectories and timepoints used in clustering [32]. To reduce the number of trajectories for clustering, senators of 100, 150, 200, 250, 300, 350, 400, 450, and 500 were defined. Time-based reduction was also performed to reduce the number of timepoints for clustering to 100, 200, or 250 timepoints and compared against models without any time reduction. kmlShape unsupervised clustering was performed with clusters of 10, 15, 20, 25, 30, 35, 40, 45, and 50 for each combination of senators and timepoint reductions. Models that failed to converge were removed from further analysis.

Clustering performance was evaluated using two internal evaluation metrics: the Calinski Harabasz score [35] and the Davies-Bouldin score [36]. To identify the optimal number of senators and clusters, the model with the highest Calinski Harabasz score and the lowest Davies-Bouldin score was evaluated using elbow method heuristics [37, 38]. The model with the highest Calinski Harabasz score and lowest Davies Bouldin score, as confirmed by the elbow method, was selected for further analysis.

### Clustering evaluation

ECG heartbeats representing the center of each cluster were visualized for the optimal model. Cluster centers were calculated as the arithmetic mean of all heartbeats assigned to a given cluster. Phenotypic features of diabetes, death, hypoglycemia, severe hypoglycemia, PR interval, QT interval, and QRS interval were calculated. Diabetes and death statuses associated with the rat from which the heartbeat originated were reported. Hypoglycemia (15–70 mg/dL, 0.83–3.89 mmol/L) and severe hypoglycemia (< 15 mg/dL, < 0.83 mmol/L) status during which the heartbeat occurred was also extracted. Finally, the status of increased PR interval (> 70 ms), QT interval (> 75.9 ms), and QRS interval (>22 ms) for each heartbeat were calculated and compared to standard interval measurements in Sprague-Dawley rats [17]. To determine if the proportion of heartbeats in each cluster differed by diabetes status, death status, hypoglycemia, severe hypoglycemia, increased PR interval, increased QT interval, or increased QRS interval, *X*^2^ test of independence were performed and evaluated at the 0.05 significance level (Python, SciPy, v1.7.3 [30]). Phenotypic features significantly different between clusters were visualized using a radar diagram. Normality was assessed using the Kolmogorov-Smirnov test (Python, SciPy, v1.7.3 [30]) and all ECG parameters were determined to be non-normally distributed (all p < 0.001). Therefore, the median and interquartile range (IQR) for the PR interval, QT interval, and QRS interval were calculated for each cluster.

### Analysis by experimental group

ECG heartbeats were categorized into eight experimental groups according to diabetes, death, and hypoglycemia or severe hypoglycemia status. Experimental groups consisted of heartbeats during hypoglycemia from non-diabetic rats that lived (H-ND-L), severe hypoglycemia from non-diabetic rats that lived (SH-ND-L), hypoglycemia from non-diabetic rats that died (H-ND-X), severe hypoglycemia from non-diabetic rats that died (SH-ND-X), hypoglycemia from diabetic rats that lived (H-D-L), severe hypoglycemia from diabetic rats that lived (SH-D-L), hypoglycemia from diabetic rats that died (H-D-X), and severe hypoglycemia from diabetic rats that died (SH-D-X). For each cluster, the percentage of heartbeats from each experimental group was calculated and scaled by experimental group. To compare the distribution of heartbeats across each cluster within a given experimental group, a heatmap of scaled percentages was displayed with a dendrogram generated by hierarchical clustering of experimental group using the Euclidean distance metric and Ward’s linkage method. The median and interquartile range (IQR) for the PR interval, QT interval, and QRS interval were calculated for each cluster for non-diabetic and diabetic rats. To determine if plasma glucose concentrations were associated with ECG changes in the PR, QT, and QRS intervals for each experimental group, we calculated the Spearman’s rank correlation (Python, SciPy, v1.7.3 [30]). Results were evaluated at the 0.05 significance level. The absolute value of the correlation coefficients were classified as negligible (0.00–0.10), weak (0.10–0.39), moderate (0.40–0.69), strong (0.70–0.89), or very strong (0.90–1.00) [39].

## Results

### Cohort characteristics

ECG heartbeats were extracted from 91 male Sprague-Dawley rats (54 non-diabetic, 37 diabetic), 24 of which died (14 non-diabetic, 10 diabetic) during insulin-induced hypoglycemia clamp experimentation. A total of 1,803 15-minute intervals that aligned with collection of blood glucose measurements were segmented across all 91 rats. Heartbeat delineation resulted in 5,719,116 heartbeats extracted across all segments for all rats (S1 Table in S1 File) that were subsequently included for clustering analysis.

### Heartbeat clustering results

**Fig 2:**
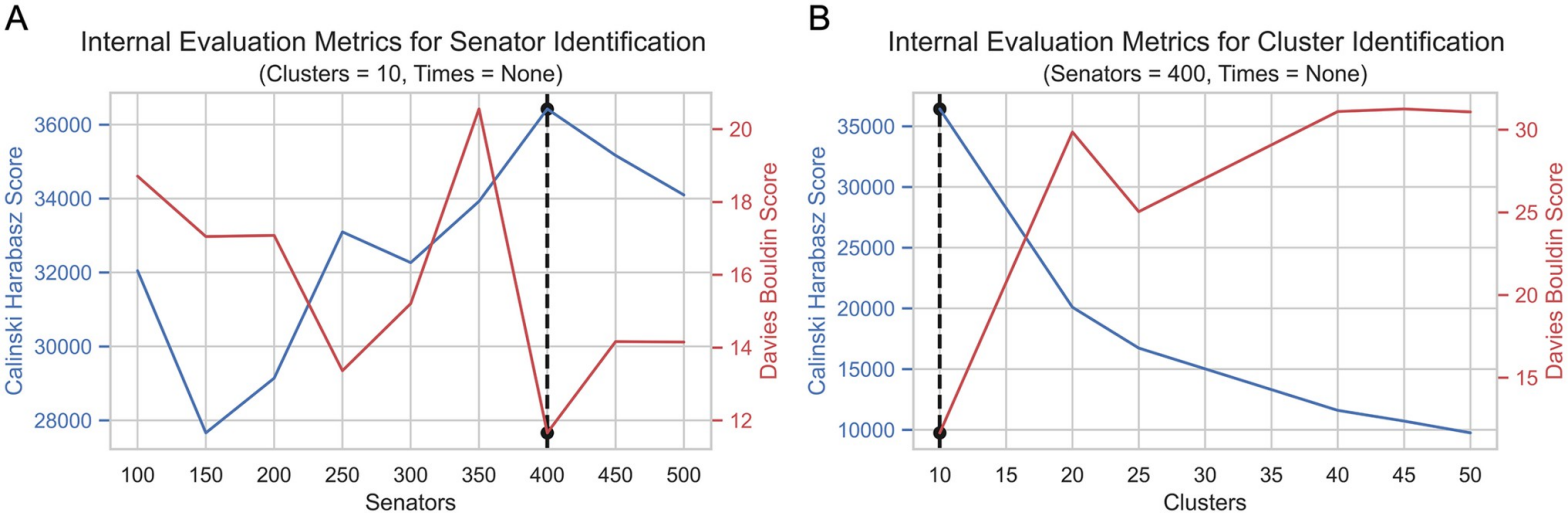
Internal evaluation metrics for senator and cluster identification. Calinski Harabasz (blue) and Davies Bouldin scores (red) for relevant unsupervised clustering models. Black dots represent the lowest Davies Bouldin score and highest Calinski Harabasz score. Dashed black line represents the senator and cluster identified as the optimal model. (A) Internal evaluation metrics for all models developed using 10 clusters and no times. 400 senators model was identified as having the highest Calinski Harabasz and lowest Davies Bouldin score using the elbow method. (B) Internal evaluation metrics for all models developed using 400 senators and no times. 10 clusters model was identified as having the highest Calinski Harabasz and lowest Davies Bouldin score using the elbow method.

Longitudinal time-series clustering using kmlShape was performed to identify distinct subgroups of heartbeats. Of the 324 models with distinct senators, times, and clusters tested, 118 models converged during clustering and were therefore used for subsequent analyses. Analysis of internal evaluation metrics revealed that the model with 400 senators, 10 clusters, and no times yielded the highest Calinski Harabasz score and the lowest Davies Bouldin score (S2 Table in S1 File). To determine if the optimal number of senators and clusters were selected, elbow method heuristics were applied and revealed an inflection point at 400 senators (Fig 2A) and 10 clusters in both the Calinski Harabasz and Davies Bouldin scores (Fig 2B). Together, these findings indicate that the kmlShape algorithm trained with 400 senators, 10 clusters, and no times yielded robust clusters. Therefore, this model was selected for further analysis.

### Interpretation of identified clusters

**Fig 3:**
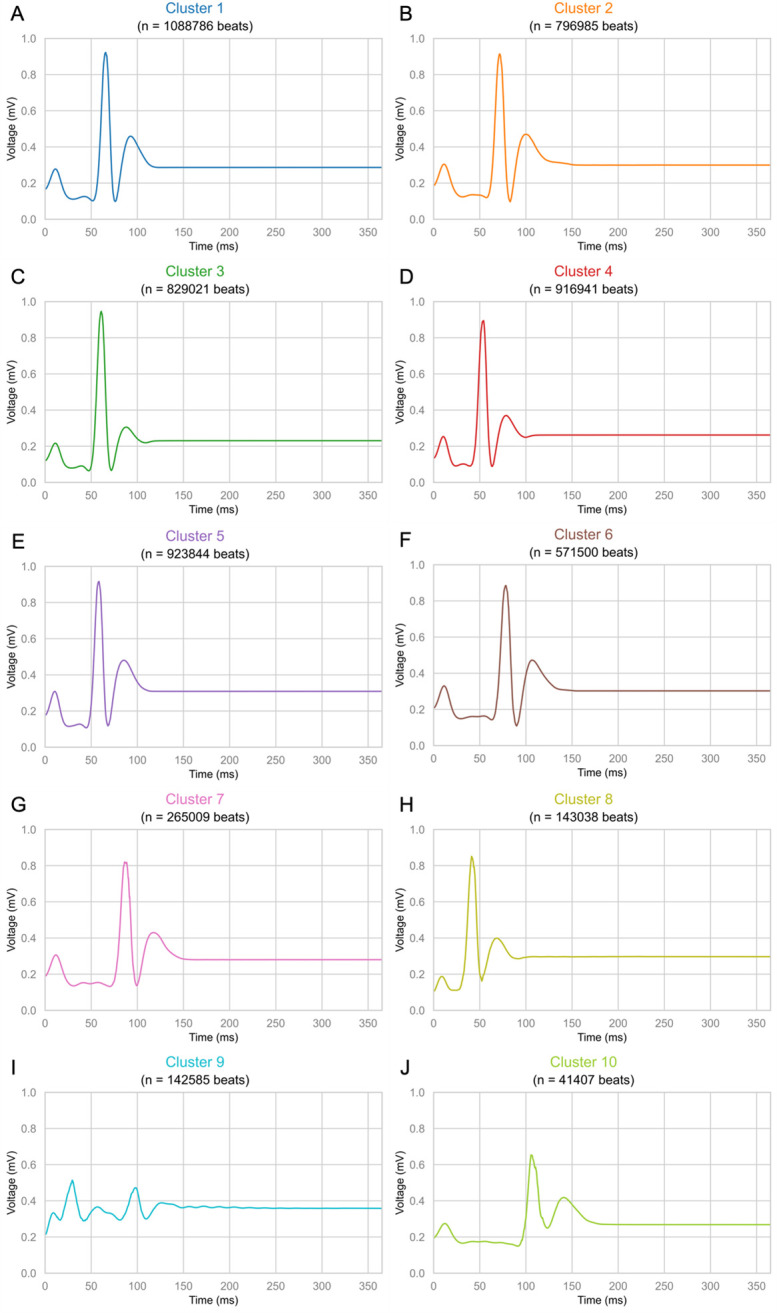
Cluster centers of the optimal model. Cluster centers representing ECG heartbeats for the unsupervised clustering model with 10 clusters, 400 senators, and no times is presented. Each of the 10 cluster centers are depicted (A–J).

**Fig 4:**
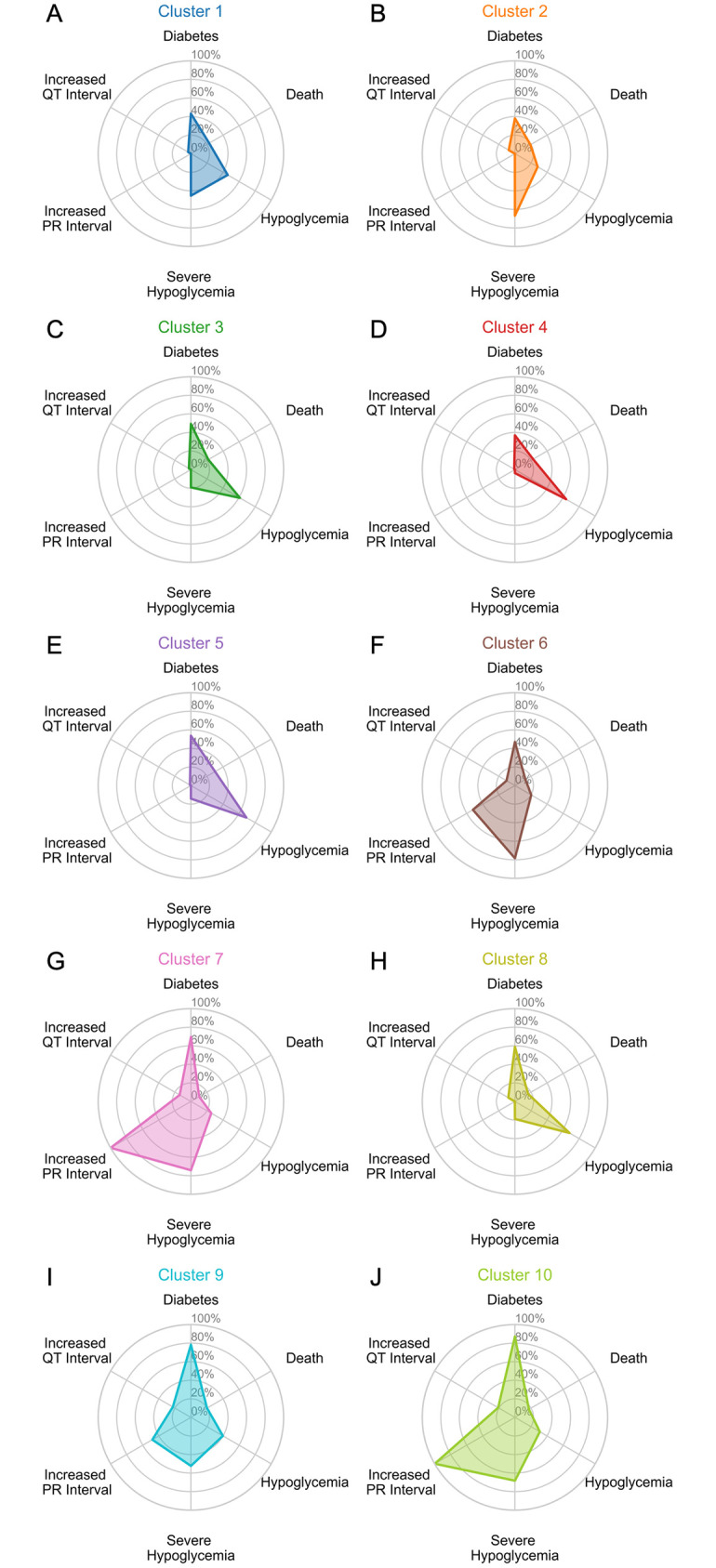
Cluster phenotypic features of the optimal model. Radar diagram representing distribution of phenotypic features for the unsupervised clustering model with 10 clusters, 400 senators, and no times is presented. The percentage of heartbeats that originated from rats with diabetes, from rats that died, that were collected during hypoglycemia, that were collected during severe hypoglycemia, that demonstrated increased PR intervals, that demonstrated increased QT intervals, and that demonstrated increased QRS intervals are depicted for each cluster (A–J).

Subgroups identified through kmlShape with 400 senators, 10 clusters, and no times were analyzed for morphology and phenotypic features. Clusters varied significantly by the proportion of heartbeats from diabetes rats, proportion of heartbeats from rats that died, heartbeats that occurred during hypoglycemia, heartbeats that occurred during severe hypoglycemia, increased PR interval length, and increased QT interval length (S3 Table in S1 File). For each identified cluster, heartbeats representing the cluster centers are depicted (Fig 3), phenotypic features are discussed (S3 Table in S1 File) and displayed as a radar diagram (Fig 4), and ECG parameters are summarized (S4 Table in S1 File).

Cluster 1 was the largest cluster (n = 1,088,786 heartbeats, 19.04%) and was characterized by normal morphology (Fig 3A), with heartbeats demonstrating variable QT interval prolongation (50.69%, S3 Table in S1 File) with a median (IQR) of 76 (7) ms (S4 Table in S1 File). Cluster 1 was the only cluster to contain similar distributions of heartbeats according to diabetes status (43.18% diabetic vs. 56.82 non-diabetic) and glycemic status (45.8% hypoglycemia vs. 45.23% severe hypoglycemia) (S3 Table in S1 File). Therefore, Cluster 1 was a non-specific cluster representing normal heartbeats regardless of diabetes and glycemic status (Fig 4A).

Clusters 3, 5, and 8 all contained a greater proportion of heartbeats that occurred during hypoglycemia (Cluster 3: 60.98%, Cluster 5: 68.94%, and Cluster 8: 67.91%) compared to severe hypoglycemia (S3 Table in S1 File). Heartbeats in Clusters 3, 5, and 8 were similarly distributed among diabetic and non-diabetic rats (S3 Table in S1 File). The centers of Clusters 3, 5, and 8 also all demonstrated normal morphology (Fig 3C, 3E and 3H), with variable increases in QT interval length (Cluster 3: 44.45%, Cluster 5: 48.90%, and Cluster 8: 43.75% S3 Table in S1 File), though the median (IQR) of all three clusters fell below the threshold for prolonged QT intervals (S3 Table in S1 File). Overall, these findings suggest that Clusters 3, 5, and 8 represent heartbeats with normal morphology that occurred during hypoglycemia independent of diabetes status (Fig 4C, 4E and 4H).

Cluster 4 was the only cluster that contained the greatest proportion of heartbeats from non-diabetic rats (63.14%) and a greater proportion of heartbeats during hypoglycemia (64.59%) compared to severe hypoglycemia (S3 Table in S1 File). The center of cluster 4 demonstrated normal morphology (Fig 4D) and had the lowest percentage of heartbeats with QT prolongation (31.57%) with the shortest median (IQR) of 73 (7) ms. This suggests that Cluster 4 represents normal heartbeats from non-diabetic rats during hypoglycemia (Fig 4D).

Clusters 2 and 6 both contained a greater proportion of heartbeats from non-diabetic rats (Cluster 2: 61.94% and Cluster 6: 53.15%) and a greater proportion of heartbeats during severe hypoglycemia (Cluster 2: 66.76% and Cluster 6: 78.31%) compared to hypoglycemia (S3 Table in S1 File). Both the centers of Clusters 2 and 6 demonstrated normal morphology (Fig 3B and 3F) with variable QT prolongation (Cluster 2: 59.98% and Cluster 6: 63.46%) and both clusters demonstrated median (IQR) QT length of 77 (7) ms (S4 Table in S1 File). Cluster 6 also demonstrated variable PR prolongation (52.34%) with a median (IQR) of 71 (4) ms (S4 Table in S1 File) and QRS prolongation (62.98%) with a median (IQR) of 23 (4) ms (S3 and S4 Tables in S1 File). Together, these findings suggest that Clusters 2 and 6 represent heartbeats from non-diabetic rats during severe hypoglycemia and can variably present with QT prolongation alone or QT prolongation in combination with PR and QRS prolongation (Fig 4B and 4F).

The final set of clusters, Clusters 7, 9, and 10, contained the highest proportion of heartbeats from diabetic rats (Cluster 7: 69.27%, Cluster 9: 78.22%, and Cluster 10: 87.03%) and a higher proportion of that occurred during severe hypoglycemia (Cluster 7: 74.08%, Cluster 9: 52.27%, Cluster 10: 68.19%) compared to hypoglycemia (S3 Table in S1 File). The center of Cluster 9 demonstrated an abnormal morphology similar to a premature ventricular contraction (Fig 3I). Cluster 7 contained a majority of heartbeats with increased QT, PR, and QRS intervals (70.16%, 99.96%, and 70.13%, respectively, S3 Table in S1 File) and demonstrated the longest median QRS length (median: 24, IQR: 5, S4 Table in S1 File). Clusters 9 and 10 contained a majority of heartbeats with increased QT intervals (Cluster 9: 70.90%, and Cluster 10: 81.46%, S3 Table in S1 File) and Cluster 10 additionally contained a majority of heartbeats with increased PR intervals (99.86%, S3 Table in S1 File) with the largest median PR interval length (median: 101, IQR: 9, S4 Table in S1 File). Together, these findings suggest that Clusters 7, 9, and 10 represent abnormal heartbeats with variably prolonged PR, QT, and QRS intervals from diabetic rats during severe hypoglycemia (Fig 4G, 4I and 4J).

### Clusters by experimental groups

**Fig 5:**
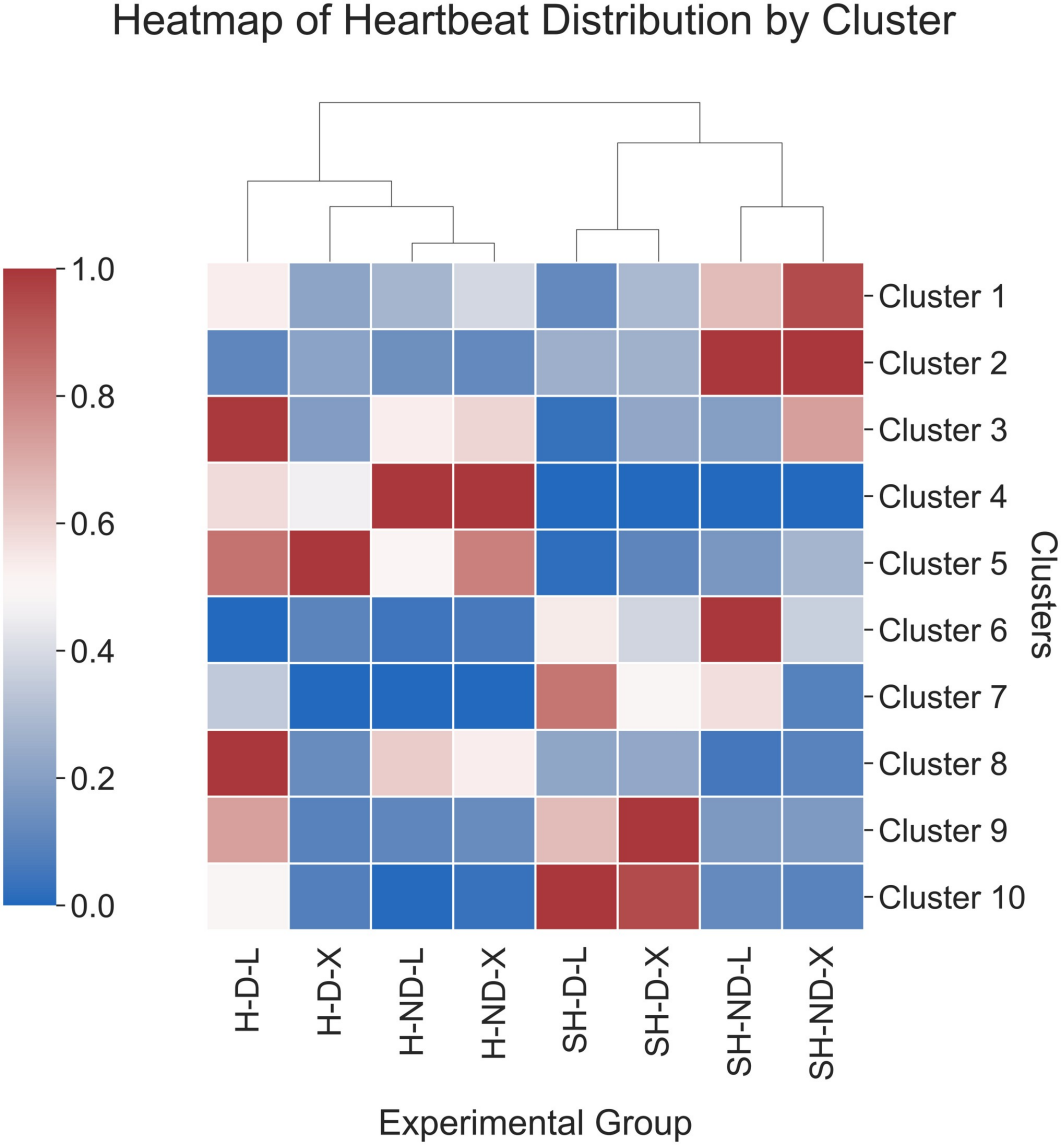
Cluster variation by experimental groups. Heatmap of heartbeat distribution across each cluster and experimental group. The color bar represents the percentage of heartbeats attributed to a given experimental group within each cluster scaled by the experimental group. Experimental groups are ordered according to similarity in hierarchical clustering demonstrated by the dendrogram at the top of the heatmap. Experimental groups were heartbeats during hypoglycemia from non-diabetic rats that lived (H-ND-L), heartbeats during severe hypoglycemia from non-diabetic rats that lived (SH-ND-L), heartbeats during hypoglycemia from non-diabetic rats that died (H-ND-X), heartbeats during severe hypoglycemia from non-diabetic rats that died (SH-ND-X), heartbeats during hypoglycemia from diabetic rats that lived (H-D-L), heartbeats during severe hypoglycemia from diabetic rats that lived (SH-D-L), heartbeats during hypoglycemia from diabetic rats that died (H-D-X), and heartbeats during severe hypoglycemia from diabetic rats that died (SH-D-X).

To compare the distribution of heartbeats across each cluster by diabetes status, death status, and hypoglycemia or severe hypoglycemia status, a heatmap of the percentage of heartbeats within a given cluster scaled by the experimental group was generated (Fig 5). The dendrogram revealed a distinct difference in cluster membership between heartbeats during hypoglycemia (left half of heatmap, Fig 5) and severe hypoglycemia (right half of heatmap, Fig 5). ECG parameters by cluster and diabetic status are also summarized (S6 Table in S1 File). The median (IQR) PR interval was similar between non-diabetic and diabetic rats for all clusters except cluster 9, which demonstrated longer PR interval in diabetic rats (S6 Table in S1 File). Similarly, the median (IQR) QRS interval was similar between non-diabetic and diabetic rats for all clusters except clusters 2, 4, 6, 7, and 8, which all demonstrated longer QRS intervals in diabetic rats (S6 Table in S1 File). In contrast, the median (IQR) QT interval was longer for diabetic rats compared to non-diabetic rats for all clusters (S6 Table in S1 File). Together, these findings suggest that diabetic and non-diabetic rats demonstrate similar ECG changes within a given cluster for PR and QRS intervals, but is more variable for QT intervals.

During hypoglycemia, non-diabetic and diabetic rats had higher percentages of heartbeats belonging to clusters with normal ECG morphology and fewer heartbeats with abnormal morphology (Figs 3 and 5). Rats in the experimental groups of H-ND-L and H-ND-X had the highest percentages of heartbeats from Cluster 4 compared to all other clusters (45.00% and 14.80%, respectively, S5 Table in S1 File). These findings suggest that non-diabetic rats demonstrate similar patterns of ECG heartbeat morphology during hypoglycemia that are independent of mortality status. In comparison, H-D-L had the highest percentages of heartbeats from Clusters 3 and 8 (38.00% and 38.20%, respectively, S5 Table in S1 File) while H-D-X had the highest percentage of heartbeats from Cluster 5 (13.00%, S5 Table in S1 File) compared to all other clusters. These findings indicate that clusters stratify rats during hypoglycemia by diabetic status.

During severe hypoglycemia, non-diabetic and diabetic rats had higher percentages of heartbeats belonging to clusters with abnormal ECG morphology (Figs 3 and 5). Notably, the distribution of heartbeats was distinct between diabetic and non-diabetic rats (right half of heatmap, Fig 5). Cluster 2, which demonstrated increased QT interval alone (Fig 3B), had the highest percentage of heartbeats among both SH-ND-L and SH-ND-X (45.00% and 9.00%, respectively, S5 Table in S1 File). In contrast, SH-D-L and SH-D-X had the highest percentage of heartbeats from clusters with abnormal morphologies. SH-D-L had the highest percentage of heartbeats from Cluster 10 (46.80%, S5 Table in S1 File), which demonstrated QT and PR prolongation (Fig 3J). In contrast, SH-D-X had the highest percentage of heartbeats from Cluster 9 (12.50%, S5 Table in S1 File), which demonstrated abnormal ECG morphology of premature ventricular contractions (Fig 3I). Together, these findings suggest that QT interval prolongation is a marker for severe hypoglycemia independent of diabetic status and that rats are more likely to demonstrate the combination of QT and PR prolongation during severe hypoglycemia.

For each experimental group, correlation between glucose values and PR, QT, and QRS intervals were assessed. A significant negative association between glucose and PR interval was observed between all experimental groups (S7 Table in S1 File). The strength of these associations were classified as weak or moderate among all hypoglycemic experimental groups, but were negligible or weak in severe hypoglycemic groups. Similarly, a significant negative association was observed between glucose and QRS intervals among all experimental groups (S7 Table in S1 File). The strength of these associations were classified as weak for all diabetic rats and were negligible or weak for all non-diabetic rats. Together, these findings suggest that as glucose levels decrease, there is a variable increase in PR and QRS intervals among all experimental groups. A significant negative association was observed between glucose and QT intervals for all experimental groups except the SH-ND-L group (S7 Table in S1 File). Glucose and QT intervals demonstrated a weak positive association in the SH-ND-L group (0.145, p < 0.001). This suggests that QT shortening during decreasingly severe hypoglycemic conditions in non-diabetic rats may confer a protective benefit against sudden death. In contrast, all other experimental groups experienced QT prolongation as glucose levels declined in the severe hypoglycemic experimental conditions.

## Discussion

Sudden death related to hypoglycemia is thought to be due to cardiac arrhythmias. A clearer understanding of the cardiac changes associated with hypoglycemia-induced mortality is needed to reduce mortality. Therefore, this work used a novel shape-based unsupervised clustering algorithm to identify distinct patterns of ECG heartbeat changes that correlated with glycemic level, diabetes status, and mortality using a rodent model. kmlShape identified 10 clusters of ECG heartbeats with robust internal evaluation metrics.

To determine if identified clusters differed significantly by phenotypic features, the proportion of heartbeats from diabetic rats, rats that died, heartbeats during hypoglycemia and severe hypoglycemia, heartbeats with increased PR intervals, and heartbeats with increased QT intervals were compared. We identified clusters that varied by diabetes and glycemic status (Fig 4). Cluster 1 included heartbeats with variable QT prolongation from diabetic and non-diabetic rats during hypoglycemia and severe hypoglycemia (Fig 4A). Abnormal prolongation of the QT interval is associated with hypoglycemia in humans [40–42] and is thought to be proarrhythmic [12, 43]. These findings suggest that while QT prolongation in rats reflects hypoglycemic-induced cardiac changes in humans, QT prolongation alone cannot explain differences in mortality rates among diabetic and non-diabetic rats, as seen in previous literature [18]. Future work is needed to understand why hypoglycemia-induced QT prolongation alone is insufficient to induce cardiac mortality.

Clusters 3, 4, 5, and 8 all represented heartbeats that occurred during hypoglycemia (Fig 4C–4E and 4H). While heartbeats assigned to Clusters 3, 5, and 8 were similarly distributed between non-diabetic and diabetic rats, heartbeats in Cluster 4 were specific to non-diabetic rats. Notably, heartbeats in Clusters 3, 5, and 8 demonstrated increased QT prolongation compared to heartbeats in Cluster 4 (S3 Table in S1 File). Taken together, these findings suggest that heartbeats from non-diabetic rats are less likely to demonstrate QT prolongation during hypoglycemia. In contrast, Clusters 2, 6, 7, 9, and 10 all represented heartbeats that occurred during severe hypoglycemia (Fig 4B, 4F, 4G, 4I and 4J). Heartbeats assigned to Clusters 2 and 6 were more common among non-diabetic rats and displayed variable QT prolongation alone or the combination of QT, PR and QRS prolongation, respectively. In contrast, heartbeats assigned to Clusters 7, 9, and 10 were more common among diabetic rats and showed consistent QT prolongation with PR prolongation or both PR and QRS prolongation. PR prolongation, also known as first-degree heart block, is associated with abnormal atrioventricular conduction and arrhythmic events [44, 45]. Prolongation of the QRS interval has been previously associated with risk for sudden death in individuals with coronary disease [46] and was identified as a subclinical marker for cardiac impairment in children with uncontrolled diabetes [47]. These findings suggest that diabetic rats are at higher risk for a combination of QT prolongation with PR prolongation or QRS prolongation. Also noteworthy was that a data-driven method blinded to diabetes type and hypoglycemia status could distinguish these subgroups. Further work is needed evaluate the ECG changes identified in this analysis and determine whether drugs targeted to modulate these ECG changes may prevent sudden cardiac death in animal models of diabetes.

To further characterize heartbeats across each cluster by experimental groups of diabetes status, death status, and glycemic status, a heatmap was generated (Fig 5). Overall, we distinguished the distribution of heartbeats across each cluster by glycemic status, which is consistent with previous findings that the extent of hypoglycemia produces different electrical cardiac changes [18]. Heartbeats occurring during hypoglycemia were characterized by distinct clusters with normal ECG morphology in both diabetic (Clusters 3, 5, and 8) and non-diabetic rats (Cluster 4). These findings suggest that a data-driven clustering approach could stratify heartbeats during hypoglycemia by diabetic status. In this study, morphologic changes during hypoglycemia in a rodent model of diabetes were not associated with increased mortality and future studies should seek to evaluate non-morphologic arrhythmias as early biomarkers for hypoglycemia-induced mortality.

During severe hypoglycemia, the heatmap revealed different cluster memberships by diabetic status and death status (Fig 5). QT prolongation was characteristic of all experimental conditions during severe hypoglycemia, supporting previous findings that severe hypoglycemia can induce abnormal QT prolongation regardless of diabetes status [40]. This analysis also found that different clusters were specific to non-diabetic and diabetic rats during severe hypoglycemia. Diabetic rats that lived demonstrated a combination of QT and PR prolongation during severe hypoglycemia (Cluster 10) while diabetic rats that died demonstrated premature ventricular contractions (Cluster 9). Together, these findings suggest that PR prolongation is more common in diabetic rats and that premature ventricular contractions may be a more precise marker for mortality in diabetic rats during severe hypoglycemia.

We also found that decreasing glucose levels were associated with increasing PT, QRS, and QT intervals among all experimental groups, with the exception of the SH-ND-L group being associated with decreasing QT intervals (S7 Table in S1 File). These findings suggest that the ability to decrease QT interval length during worsening severe hypoglycemia may confer protection against sudden cardiac death. Future studies should seek to evaluate the mechanistic role of QT shortening in preventing hypoglycemia-induced cardiac arrhythmias.

There are several limitations to this work. In this analysis, insulin-induced mortality rates were similar between non-diabetic and diabetic rats. Retrospective analysis with large sample sizes demonstrated significant increase in mortality in diabetic rats compared to non-diabetic rats [18]. Mortality rates in this manuscript do not reflect mortality rates from the original experiments [3, 18, 19, 24] because only non-diabetic and diabetic rats from control conditions were included in analysis. Notably, non-diabetic rats are noted to have diverse rates of insulin-induced mortality, ranging from 14%–47% [3, 18, 19, 24], that may be attributed to variability in hypokalemia, neuroglycopenia, or sympathoadrenal responses that were not assessed in this analysis. Future studies should seek to explore the role of these additional factors in insulin-induced mortality.

A novel methodology to detect QRS complexes, P waves, and T waves was developed because other methods failed to delineate these waveforms, likely due to the lack of Q waves, shorter RR intervals, and immediate onset of the T-wave following QRS onset seen in rat ECGs. While this methodology was sufficient to detect ECG heartbeats, rhythm-based arrhythmias were not identified. Future studies should seek to utilize data-driven tools to evaluate rhythm-based arrhythmias in hypoglycemia-induced mortality. kmlShape was used to perform unsupervised clustering and several training parameters were implemented to limit computational complexity. While the combination of senators, clusters, and times yielded robust evaluation metrics, future studies should explore alternative unsupervised clustering methods that require limited considerations for computational complexity. Visualization of cluster centers was generated by calculating the arithmetic mean of all heartbeats assigned to a given cluster. Variation in the time and voltage of the T-wave offset resulted in a higher baseline after the T-wave compared to the P-wave onset.

## Conclusion

This study provides the first data-driven characterization of ECG heartbeats in a rodent model of diabetes during hypoglycemia. The use of kmlShape provides a novel morphology-based assessment of electrical cardiac changes during hypoglycemia. These findings suggest that data-driven methods can stratify heartbeats by glycemic level and diabetes status and the more frequent combination of PR prolongation and QT prolongation observed in diabetic rats should be explored in analysis of human electrocardiograms. Overall, these findings pave the way for the identification of sequences of conduction abnormalities that lead to hypoglycemia-induced deaths.

## Supporting information

S1 File (DOCX)
