# Supplementary material for: Detecting hypoglycemia-induced electrocardiogram changes in a rodent model of type 1 diabetes using shape-based clustering
Source: PLoS One. 2023 May 18;18(5):e0284622. doi: 10.1371/journal.pone.0284622 (PMC10194943; doi:10.1371/journal.pone.0284622)
Supplement: S1 File — (DOCX) [file pone.0284622.s001.docx]

**SUPPLEMENTARY MATERIALS**

**S1 Methods. Additional Details on Materials and Methods - Preprocessing.** Further description of the ECG preprocessing methods, including ECG filtering and heartbeat extraction.

**S1 Figure. ECG Filtering and Delineation.** (A) ECG waveform filtering process before and after removal of baseline wander (top) and powerline interference (bottom). (B) ECG waveform heartbeat delineation for identifying R peaks, P peaks, and T peaks.

**S1 Table. Heartbeats Extracted Across All Segments and Rats.** Number of heartbeats for each rat (left) and segment (right) used in clustering analysis.

**S2 Table. Top Performing Models.** The senators, times, and clusters used to train the top 5 models with the highest Calinski Harabasz score and the 5 models with the lowest Davies-Bouldin score are presented.

**S3 Table. Distribution of Phenotypic Features by Cluster.** The percentage and number of heartbeats assigned to a give cluster relative to the total number of heartbeats included in analysis. Phenotypic features, including the number and percentage of heartbeats that originated from rats with diabetes, from rats that died, that were collected during hypoglycemia, that were collected during severe hypoglycemia, that demonstrated increased PR intervals, that demonstrated increased QT intervals, and that demonstrated increased QRS intervals relative to each cluster. Significant differences in phenotypic features are calculated using a X2 test of independence and evaluated at the 0.05 significance level.

**S4 Table. Distribution of ECG Parameters by Cluster.** The median and interquartile range (IQR) of the PR interval, QT interval, and QRS interval of all heartbeats assigned to each cluster are summarized. All values are reported in milliseconds.

**S5 Table. Percentage of Heartbeats by Experimental Group**. The percentage of heartbeats for each experimental group relative to the total number of heartbeats in each cluster. Experimental groups were heartbeats during hypoglycemia from non-diabetic rats that lived (H-ND-L), heartbeats during severe hypoglycemia from non-diabetic rats that lived (SH-ND-L), heartbeats during hypoglycemia from non-diabetic rats that died (H-ND-X), heartbeats during severe hypoglycemia from non-diabetic rats that died (SH-ND-X), heartbeats during hypoglycemia from diabetic rats that lived (H-D-L), heartbeats during severe hypoglycemia from diabetic rats that lived (SH-D-L), heartbeats during hypoglycemia from diabetic rats that died (H-D-X), and heartbeats during severe hypoglycemia from diabetic rats that died (SH-D-X).

**S6 Table. Distribution of ECG Parameters by Cluster and Diabetes Status**. The median and interquartile range (IQR) of the PR interval, QT interval, and QRS interval of all heartbeats assigned to each cluster are summarized for each cluster and diabetes status. All values are reported in milliseconds.

**S7 Table.** **Association Between Glucose and ECG Parameters by Experimental Group**. Association between glucose concentrations and PR, QT, and QRS intervals were assessed for each experimental group using the Spearman’s rank correlation. Experimental groups were heartbeats during hypoglycemia from non-diabetic rats that lived (H-ND-L), heartbeats during severe hypoglycemia from non-diabetic rats that lived (SH-ND-L), heartbeats during hypoglycemia from non-diabetic rats that died (H-ND-X), heartbeats during severe hypoglycemia from non-diabetic rats that died (SH-ND-X), heartbeats during hypoglycemia from diabetic rats that lived (H-D-L), heartbeats during severe hypoglycemia from diabetic rats that lived (SH-D-L), heartbeats during hypoglycemia from diabetic rats that died (H-D-X), and heartbeats during severe hypoglycemia from diabetic rats that died (SH-D-X).

**S1 Methods: Additional Details on Materials and Methods - Preprocessing**

**Preprocessing**:

*ECG Filtering*: Baseline wander is a low-frequency artifact in electrocardiograms (ECGs) that arises from movements, imperfect electrode contact, and respiration. Notch filters (Python Heart Rate Analysis Toolkit, v0.8.1) with frequency cutoffs of 0.001, 0.005, 0.01, 0.05, 0.1, and 0.5 were tested. A cutoff of 0.1 was visually identified as the optimal threshold to remove baseline wander and preserve ECG architecture. Powerline interference is a harmonic artifact in ECGs that is caused by electromagnetic interference from machinery and can obscure the P and T waves. A low-pass Butterworth filter, high-pass Butterworth filter, and band-pass Butterworth filter (Python Heart Rate Analysis Toolkit, v0.8.1) with a lower frequency cutoff of 0.5 – 2.0 and an upper frequency of 30.0-100.0 were tested. The band-pass Butterworth filter with a lower frequency of 1.5 and a higher frequency of 65.0 was visually identified as the optimal cutoff to remove powerline interference.

*Heartbeat Segmentation*: Several methodologies were tested for R wave delineation. The Pan-Tompkins Algorithm, Christov Segmenter, Hamilton Segmenter, and Wavelet-based ECG delineation failed to consistently detect RS complexes, likely due to the lack of Q waves, shorter RR interval, and immediate onset of the T wave following the QRS complex in rat ECGs. Therefore, a novel approach was created to detect the R waves. ECG data was scaled from 0 – 1.0 mV in intervals of 500 ms to account for the variability of peak sizes. SciPy peak-finding algorithm with prominences of 0.2 – 1.0, widths of 0 – 50, and relative heights of 0.2 – 1.0 were tested. Prominence of 0.35, width of 25, and relative height of 0.3 were identified as the optimal parameters to detect R waves. Given that rat T waves immediately follow RS complexes, wider T waves may be incorrectly identified as R waves. Prominences and widths of ECG segments that contained T waves incorrectly defined as R waves were found to have a standard deviation greater than 0.15. The incorrectly identified waves were filtered by removing peaks with prominence or width below one standard deviation of the mean.

P and T Wave Delineation: To identify a P wave at Ri, SciPy peak-finding algorithm with a sliding window method starting from Ri continuing to Ri-1 was used to identify the most prominent peak within the search distance. SciPy peak width finding algorithm was used to determine the onset of the identified P wave. To identify a T wave at Ri, SciPy peak-finding algorithm with a sliding window method starting from Ri continuing to Ri+1 was used to identify the most prominent peak within the search distance. SciPy peak width finding algorithm was used to determine the offset of the identified T wave.

**S1 Figure: ECG Filtering and Delineation**


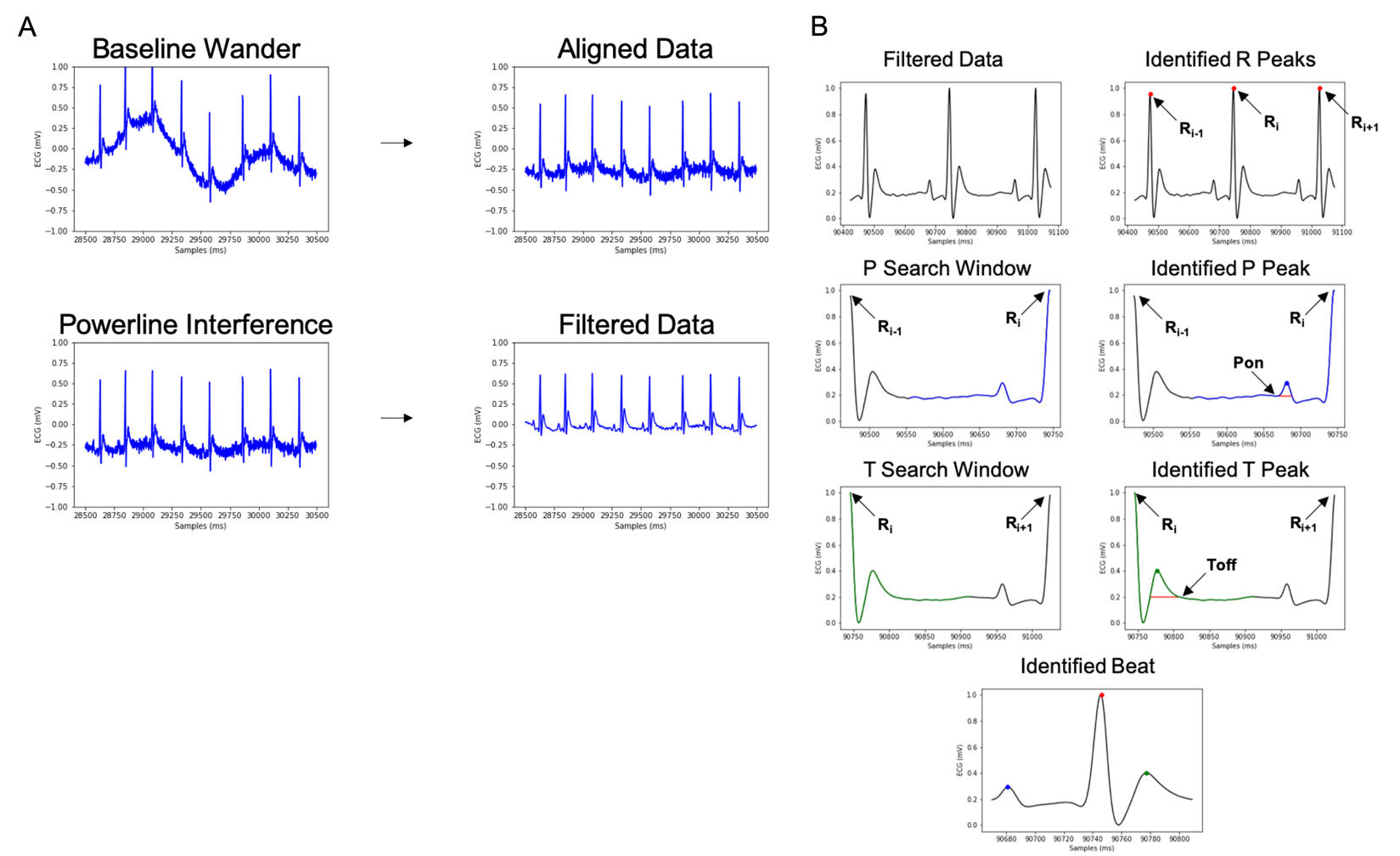


**S1 Table: Heartbeats Extracted Across All Segments and Rats**

| **Heartbeats Per Rat** | |  | **Heartbeats Per Condition** | |
| --- | --- | --- | --- | --- |
| Rat ID | Heartbeat Counts |  | Insulin Clamp Condition | Heartbeat Counts |
| 1 | 52075 |  | Insulin Start | 422374 |
| 2 | 92556 |  | Insulin 15 Minutes | 368539 |
| 3 | 32950 |  | Insulin 30 Minutes | 366568 |
| 4 | 92572 |  | Insulin 45 Minutes | 339992 |
| 5 | 72771 |  | Insulin 60 Minutes | 298118 |
| 6 | 99827 |  | Insulin 75 Minutes | 260821 |
| 7 | 74219 |  | Insulin 90 Minutes | 182103 |
| 8 | 44002 |  | Insulin 105 Minutes | 150525 |
| 9 | 77425 |  | Insulin 120 Minutes | 136904 |
| 10 | 50408 |  | Insulin 135 Minutes | 122640 |
| 11 | 57590 |  | Insulin 150 Minutes | 106553 |
| 12 | 89260 |  | Insulin 165 Minutes | 80006 |
| 13 | 71426 |  | Insulin 180 Minutes | 68908 |
| 14 | 89146 |  | Insulin 195 Minutes | 54977 |
| 15 | 68606 |  | Insulin 210 Minutes | 36454 |
| 16 | 41840 |  | Insulin 225 Minutes | 34440 |
| 17 | 50739 |  | Insulin 240 Minutes | 27929 |
| 18 | 53721 |  | Insulin 255 Minutes | 13038 |
| 19 | 72241 |  | Insulin 270 Minutes | 8822 |
| 20 | 56907 |  | Insulin 285 Minutes | 5027 |
| 21 | 59433 |  | Insulin 300 Minutes | 3358 |
| 22 | 56453 |  | Insulin 315 Minutes | 2638 |
| 23 | 51950 |  | Insulin 330 Minutes | 2823 |
| 24 | 50268 |  | Insulin 345 Minutes | 1687 |
| 25 | 62793 |  | Insulin 360 Minutes | 2750 |
| 26 | 108438 |  | Insulin 375 Minutes | 2262 |
| 27 | 60860 |  | Insulin 390 Minutes | 1951 |
| 28 | 57797 |  | Insulin 405 Minutes | 2048 |
| 29 | 57241 |  | Insulin 420 Minutes | 1540 |
| 30 | 69074 |  | Severe Hypoglycemia Start | 298216 |
| 31 | 45048 |  | Severe Hypoglycemia 15 Minutes | 258662 |
| 32 | 58762 |  | Severe Hypoglycemia 30 Minutes | 248940 |
| 33 | 87652 |  | Severe Hypoglycemia 45 Minutes | 240903 |
| 34 | 51948 |  | Severe Hypoglycemia 60 Minutes | 232287 |
| 35 | 25764 |  | Severe Hypoglycemia 75 Minutes | 214960 |
| 36 | 98640 |  | Severe Hypoglycemia 90 Minutes | 212592 |
| 37 | 57662 |  | Severe Hypoglycemia 105 Minutes | 203867 |
| 38 | 36693 |  | Severe Hypoglycemia 120 Minutes | 190037 |
| 39 | 65984 |  | Severe Hypoglycemia 135 Minutes | 182543 |
| 40 | 68122 |  | Severe Hypoglycemia 150 Minutes | 173418 |
| 41 | 39608 |  | Severe Hypoglycemia 165 Minutes | 145573 |
| 42 | 53666 |  | Severe Hypoglycemia 180 Minutes | 5534 |
| 43 | 57935 |  | Severe Hypoglycemia 195 Minutes | 5789 |
| 44 | 55926 |  |  |  |
| 45 | 23914 |  |  |  |
| 46 | 58043 |  |  |  |
| 47 | 49161 |  |  |  |
| 48 | 69318 |  |  |  |
| 49 | 92068 |  |  |  |
| 50 | 93491 |  |  |  |
| 51 | 48209 |  |  |  |
| 52 | 62873 |  |  |  |
| 53 | 77638 |  |  |  |
| 54 | 75664 |  |  |  |
| 55 | 55403 |  |  |  |
| 56 | 66659 |  |  |  |
| 57 | 45712 |  |  |  |
| 58 | 17744 |  |  |  |
| 59 | 83217 |  |  |  |
| 60 | 97218 |  |  |  |
| 61 | 63856 |  |  |  |
| 62 | 70063 |  |  |  |
| 63 | 41722 |  |  |  |
| 64 | 59830 |  |  |  |
| 65 | 43616 |  |  |  |
| 66 | 59477 |  |  |  |
| 67 | 34090 |  |  |  |
| 68 | 68008 |  |  |  |
| 69 | 75845 |  |  |  |
| 70 | 71752 |  |  |  |
| 71 | 100058 |  |  |  |
| 72 | 76470 |  |  |  |
| 73 | 59049 |  |  |  |
| 74 | 74232 |  |  |  |
| 75 | 61458 |  |  |  |
| 76 | 49376 |  |  |  |
| 77 | 61267 |  |  |  |
| 78 | 20555 |  |  |  |
| 79 | 47534 |  |  |  |
| 80 | 60882 |  |  |  |
| 81 | 67617 |  |  |  |
| 82 | 57695 |  |  |  |
| 83 | 36293 |  |  |  |
| 84 | 75835 |  |  |  |
| 85 | 99403 |  |  |  |
| 86 | 77154 |  |  |  |
| 87 | 66300 |  |  |  |
| 88 | 63158 |  |  |  |
| 89 | 46815 |  |  |  |
| 90 | 95558 |  |  |  |
| 91 | 39818 |  |  |  |

**S2 Table: Top Performing Models**

|  | | **Model Information** | | | **Scores** | |
| --- | --- | --- | --- | --- | --- | --- |
|  |  | Times | Senator | Cluster | Calinski Harabasz | Davies Bouldin |
| **Top-5 Models** | Highest  Calinski Harabasz  Scores | 1 | 400 | 10 | 36421.858 | 11.65866834 |
|  |  | 1 | 450 | 10 | 35165.58381 | 14.16385762 |
|  |  | 1 | 500 | 10 | 34096.96754 | 14.15157496 |
|  |  | 1 | 350 | 10 | 33923.99897 | 20.55298537 |
|  |  | 1 | 250 | 10 | 33096.2586 | 13.36486449 |
|  | Lowest  Davies Bouldin Scores | 1 | 400 | 10 | 36421.858 | 11.65866834 |
|  |  | 1 | 250 | 10 | 33096.2586 | 13.36486449 |
|  |  | 1 | 500 | 10 | 34096.96754 | 14.15157496 |
|  |  | 1 | 450 | 10 | 35165.58381 | 14.16385762 |
|  |  | 1 | 300 | 10 | 32265.49183 | 15.20758637 |

**S3 Table: Distribution of Phenotypic Features by Cluster**

|  | **Observations** | **Diabetes** | **Death** | **Hypoglycemia** | **Severe Hypoglycemia** | **Increased**  **PR Interval** | **Increased**  **QT Interval** | **Increased**  **QRS Interval** |
| --- | --- | --- | --- | --- | --- | --- | --- | --- |
| **Cluster 1** | 19.04%  (n = 1088786) | 43.18%  (n = 470186) | 22.7%  (n = 247169) | 45.8%  (n = 498648) | 45.23%  (n = 492471) | 0.02%  (n = 193) | 50.69%  (n = 551927) | 32.62%  (n = 355197) |
| **Cluster 2** | 13.94%  (n = 796985) | 38.06%  (n = 303334) | 19.34%  (n = 154162) | 28.27%  (n = 225289) | 66.76%  (n = 532102) | 0.08%  (n = 642) | 59.98%  (n = 478044) | 48.91%  (n = 389766) |
| **Cluster 3** | 14.5%  (n = 829021) | 49.18%  (n = 407717) | 21.62%  (n = 179220) | 60.98%  (n = 505560) | 19.16%  (n = 158818) | 0.00%  (n = 33) | 44.45%  (n = 368541) | 30.46%  (n = 252547) |
| **Cluster 4** | 16.03%  (n = 916941) | 36.86%  (n = 337951) | 22.58%  (n = 207034) | 63.59%  (n = 583102) | 3.77%  (n = 34575) | 0.01%  (n = 69) | 31.57%  (n = 289467) | 12.99%  (n = 119090) |
| **Cluster 5** | 16.15%  (n = 923844) | 53.59%  (n = 495083) | 30.06%  (n = 277670) | 68.94%  (n = 636883) | 14.12%  (n = 130425) | 0.03%  (n = 296) | 48.90%  (n = 451720) | 21.55%  (n = 199124) |
| **Cluster 6** | 9.99%  (n = 571500) | 46.85%  (n = 267771) | 13.05%  (n = 74579) | 20.3%  (n = 116020) | 78.31%  (n = 447534) | 52.34%  (n = 299143) | 63.46%  (n = 362669) | 62.98%  (n = 359920) |
| **Cluster 7** | 4.63%  (n = 265009) | 69.27%  (n = 183584) | 10.31%  (n = 27316) | 25.32%  (n = 67104) | 74.08%  (n = 196311) | 99.96%  (n = 264916) | 70.16%  (n = 185941) | 70.13%  (n = 185858) |
| **Cluster 8** | 2.5%  (n = 143038) | 58.76%  (n = 84051) | 16.33%  (n = 23352) | 67.91%  (n = 97135) | 18.89%  (n = 27017) | 0.13%  (n = 183) | 43.75%  (n = 62586) | 26.07%  (n = 37284) |
| **Cluster 9** | 2.49%  (n = 142585) | 78.22%  (n = 111525) | 20.01%  (n = 28538) | 39.81%  (n = 56764) | 52.27%  (n = 74530) | 48.05%  (n = 68514) | 70.90%  (n = 101093) | 56.24%  (n = 80184) |
| **Cluster 10** | 0.72%  (n = 41407) | 87.03%  (n = 36037) | 17.4%  (n = 7206) | 30.96%  (n = 12818) | 68.19%  (n = 28234) | 99.86%  (n = 41351) | 81.46%  (n = 33729) | 56.77%  (n = 23507) |
| **p-value** |  | < 0.001 | < 0.001 | < 0.001 | < 0.001 | < 0.001 | < 0.001 | < 0.001 |

**S4 Table: Distribution of ECG Parameters by Cluster**

|  | PR Interval  Median (IQR) | QT Interval  Median (IQR) | QRS Interval  Median (IQR) |
| --- | --- | --- | --- |
| Cluster 1 | 59 (3) | 76 (7) | 21 (3) |
| Cluster 2 | 65 (3) | 77 (7) | 22 (4) |
| Cluster 3 | 54 (2) | 75 (7) | 21 (4) |
| Cluster 4 | 47 (3) | 73 (7) | 19 (3) |
| Cluster 5 | 51 (2) | 75 (8) | 20 (4) |
| Cluster 6 | 71 (4) | 77 (7) | 23 (4) |
| Cluster 7 | 80 (5) | 78 (7) | 24 (5) |
| Cluster 8 | 35 (5) | 74 (8) | 20 (5) |
| Cluster 9 | 41 (68) | 79 (10) | 23 (8) |
| Cluster 10 | 101 (9) | 80 (7) | 23 (5) |

**S5 Table: Percentage of Heartbeats by Experimental Group**

|  | H-ND-L | SH-ND-L | H-ND-X | SH-ND-X | H-D-L | SH-D-L | H-D-X | SH-D-X |
| --- | --- | --- | --- | --- | --- | --- | --- | --- |
| Cluster 1 | 13.80% | 30.80% | 5.80% | 8.60% | 26.10% | 6.60% | 4.60% | 3.80% |
| Cluster 2 | 7.90% | 45.00% | 2.00% | 9.00% | 15.30% | 12.80% | 4.50% | 3.50% |
| Cluster 3 | 24.90% | 11.50% | 8.90% | 6.80% | 38.00% | 2.50% | 4.30% | 3.10% |
| Cluster 4 | 45.00% | 3.30% | 14.80% | 0.90% | 27.30% | 1.20% | 7.20% | 0.20% |
| Cluster 5 | 23.70% | 10.40% | 12.00% | 3.10% | 34.30% | 2.00% | 13.00% | 1.60% |
| Cluster 6 | 3.80% | 44.90% | 1.10% | 3.80% | 12.40% | 25.80% | 3.30% | 4.90% |
| Cluster 7 | 1.80% | 27.10% | 0.20% | 1.60% | 21.20% | 39.60% | 2.20% | 6.20% |
| Cluster 8 | 28.40% | 5.50% | 8.00% | 1.60% | 38.20% | 11.50% | 3.60% | 3.10% |
| Cluster 9 | 6.60% | 10.60% | 2.10% | 2.30% | 31.20% | 31.30% | 3.20% | 12.50% |
| Cluster 10 | 2.10% | 8.50% | 0.70% | 1.60% | 25.20% | 46.80% | 3.20% | 11.80% |

**S6 Table: Distribution of ECG Parameters by Cluster and Diabetes Status**

|  |  | PR Interval  Median (IQR) | QT Interval  Median (IQR) | QRS Interval  Median (IQR) |
| --- | --- | --- | --- | --- |
| Cluster 1 | Non-Diabetic | 59 (2) | 75 (6) | 21 (3) |
|  | Diabetic | 59 (3) | 77 (7) | 22 (4) |
| Cluster 2 | Non-Diabetic | 65 (3) | 76 (6) | 22 (4) |
|  | Diabetic | 64 (3) | 80 (9) | 24 (7) |
| Cluster 3 | Non-Diabetic | 54 (3) | 73 (9) | 20 (5) |
|  | Diabetic | 54 (2) | 76 (6) | 21 (4) |
| Cluster 4 | Non-Diabetic | 48 (3) | 71 (8) | 19 (2) |
|  | Diabetic | 47 (3) | 76 (5) | 21 (3) |
| Cluster 5 | Non-Diabetic | 51 (2) | 75 (7) | 20 (4) |
|  | Diabetic | 51 (3) | 76 (7) | 20 (4) |
| Cluster 6 | Non-Diabetic | 71 (4) | 76 (6) | 23 (4) |
|  | Diabetic | 71 (4) | 80 (8) | 25 (6) |
| Cluster 7 | Non-Diabetic | 79 (5) | 76 (7) | 23 (4) |
|  | Diabetic | 80 (5) | 80 (8) | 25 (5) |
| Cluster 8 | Non-Diabetic | 35 (4) | 72 (5) | 18 (3) |
|  | Diabetic | 35 (6) | 77 (8) | 21 (5) |
| Cluster 9 | Non-Diabetic | 27 (65) | 77 (12) | 23 (12) |
|  | Diabetic | 85 (68) | 79 (10) | 23 (8) |
| Cluster 10 | Non-Diabetic | 102 (11) | 77 (8) | 23 (6) |
|  | Diabetic | 101 (9) | 80 (8) | 23 (5) |

**S7 Table: Association Between Glucose and ECG Parameters by Experimental Group**

|  | PR Interval | | | QT Interval | | | QRS Interval | | | |
| --- | --- | --- | --- | --- | --- | --- | --- | --- | --- | --- |
|  | Stat | P-value | Interpretation | Stat | P-value | Interpretation | Stat | P-value | Interpretation |  |
| H-ND-L | -0.446 | <0.001 | Moderate | -0.433 | <0.001 | Moderate | -0.360 | <0.001 | Weak |  |
| H-ND-X | -0.311 | <0.001 | Weak | -0.355 | <0.001 | Weak | -0.086 | <0.001 | Negligible |  |
| H-D-L | -0.400 | <0.001 | Moderate | -0.258 | <0.001 | Weak | -0.188 | <0.001 | Weak |  |
| H-D-X | -0.411 | <0.001 | Moderate | -0.200 | <0.001 | Weak | -0.225 | <0.001 | Weak |  |
| SH-ND-L | -0.138 | <0.001 | Weak | 0.145 | <0.001 | Weak | -0.061 | <0.001 | Negligible |  |
| SH-ND-X | -0.085 | <0.001 | Negligible | -0.016 | <0.001 | Negligible | -0.282 | <0.001 | Weak |  |
| SH-D-L | -0.114 | <0.001 | Weak | -0.212 | <0.001 | Weak | -0.322 | <0.001 | Weak |  |
| SH-D-X | -0.073 | <0.001 | Negligible | -0.060 | <0.001 | Negligible | -0.236 | <0.001 | Weak |  |
